# Measurement of Microsphere Concentration Using a Flow Cytometer with Volumetric Sample Delivery

**DOI:** 10.6028/jres.119.027

**Published:** 2014-12-29

**Authors:** Lili Wang, Yu-Zhong Zhang, Steven Choquette, AK Gaigalas

**Affiliations:** 1 National Institute of Standards and Technology, Gaithersburg, MD 20899; 2 Life Technologies Corp., Eugene, OR 97402

**Keywords:** attune flow cytometer, concentration, microspheres

## Abstract

Microsphere concentrations are needed to assign equivalent reference fluorophores (ERF) units to microspheres used in quantitative flow cytometry. A flow cytometer with a syringe based sample delivery system was evaluated for the measurement of the concentration of microspheres contained in a vial of lyophilized microspheres certified by BD Biosciences to contain 50,600 microspheres. The concentration was measured by counting the number of microspheres contained in the volume delivered by the flow cytometer and dividing the number by the volume. The syringe volume was calibrated both in the delivery and draw modes, and the results of the volume calibration were summarized by two calibration lines. The delivered volume was obtained by dividing the number of recorded events by the concentration of microsphere count standard in the sample tube. The draw volume was obtained by weighting the sample tube before and after the draw. The slope of the draw volume calibration line was equal to 1.00 with an offset of −13 µL. The slope of the delivered volume calibration was 0.93 suggesting a systematic volume-dependent bias, which can be rationalized as an effect of suspension flow in capillaries. When the sample volume was set to values between 150 µL and 300 µL, both calibration curves gave similar results suggesting that a good estimate of the true delivered volume can be obtained by subtracting 13 µL from the delivered volume indicated by the syringe settings. The number of microspheres in the volume was obtained by passing the suspension contained in the volume through a laser beam and counting the number of events in which the signals from the scattering and fluorescence detectors exceeded threshold values.

Measurements were performed with the lyophilized microspheres made by BD Biosciences and fluorescein microspheres (expired reference material RM 8640) in three buffers: a phosphate buffer saline (PBS), a buffer containing PBS and 0.05 % BSA (bovine serum albumin) by mass, and a buffer containing PBS and 0.05 % TWEEN 20 detergent solution (P1379 Sigma-Aldrich) by mass. It was found that the concentration of count standard was significantly higher in the PBS+BSA buffer relative to the value obtained in PBS buffer. Values for PBS+0.05 % TWEEN 20 buffer were intermediate. The effect of buffer on the measured microsphere concentration was reported previously.

The suggested procedure for the measurement of the concentration of microspheres with the flow cytometer is to use PBS+0.05 % BSA buffer, accumulate data for a delivered volume of 150 µL to 300 µL, and reduce the indicated delivered volume by 13 µL when performing the concentration calculation. The procedure was tested on a mixture of lyophilized microspheres and RM 8640 microspheres. The resulting lyophilized microsphere concentration was consistent with the certified value. The RM 8640 concentration determined using the suggested procedure was consistent with the concentration value determined using the relative method with the lyophilized microspheres as the reference. The uncertainties, obtained from one standard deviation of repeated measurements, were about 4 %.

## 1. Introduction

The accurate measurement of the concentration of specific cell types in peripheral blood is an important goal [1, 2]. In addition, quantitative flow cytometer measurements require microsphere standards whose production requires the measurement of microsphere concentration [3]. Therefore the development of an accurate and convenient method for the measurement of microparticle concentration is an important effort. The Attune acoustic focusing flow cytometer^1^ provides a means to measure micro particle concentration [4]. The Attune is equipped with a syringe pump which is used to deliver a measured sample suspension through the focused laser beam(s) interrogation region. The fluorescence and light scattering pulses from the microspheres are detected and stored as events. Therefore the number of detected events can be associated with a sample volume used to generate the events, and the microsphere concentration can be estimated by dividing the number of events by the associated sample volume. The purpose of this manuscript is to identify some of the uncertainties in the measurement of concentration using the Attune flow cytometer. The uncertainty results from Poisson counting statistics, the variability of the software gates used to define events, pipetting, settling of the microspheres in the sample vial, adsorption of microspheres on the walls of the container, and uncertainty in determining the volume. The sample suspensions were obtained by diluting tubes of Trucount microsphere count standard (BD Biosciences catalog number 340334) with phosphate buffer saline (PBS) and other buffers. The Trucount tube is certified by BD Biosciences to contain approximately 50,600 (uncertainty unspecified) microspheres [5]. The concentration measured with the Attune flow cytometer was consistent with the expected value if a PBS+0.05 % BSA was used as the buffer. Several systematic biases were identified which could affect the value of the measured concentration.

## 2. Experimental Procedure

**Fig. 1:**
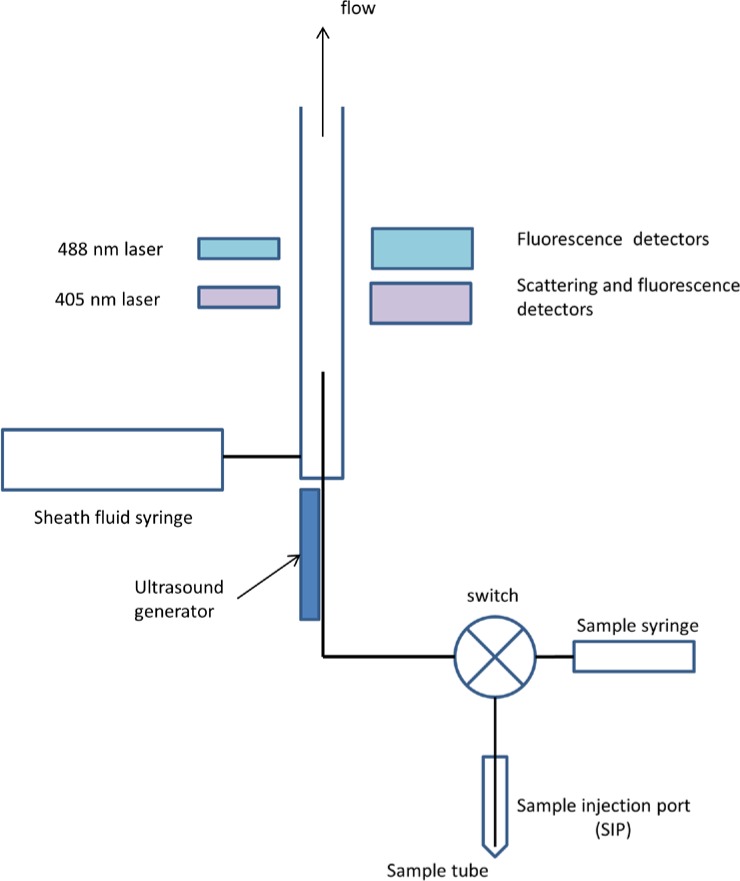
A schematic of the Attune acoustic focusing flow cytometer fluidics and detector layout. The various components are not to scale, and the fluidic components needed for washing and other instrument support functions were omitted. The user places a tube containing the sample in the “sample injection port” (SIP), sets the measurement conditions (flow rate and total sampled volume), and starts the measurement. The Attune first draws a specified amount of sample from the tube in the SIP, and then forces another specified amount of the sample in the syringe through the capillary and the detection region. The instrument then purges the left over sample, and prepares the fluidics for another measurement sequence.

**Fig. 2:**
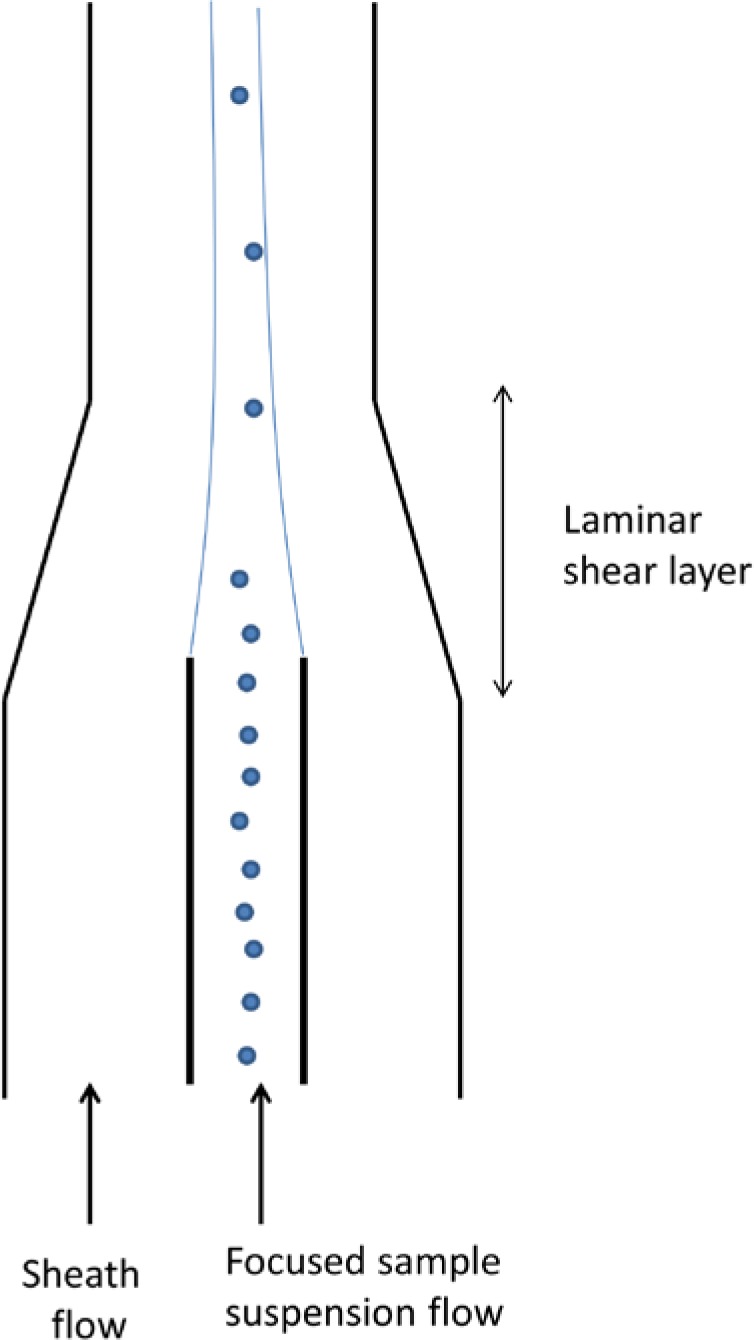
A schematic of the part of the Attune fluidics system where the sample suspension flow and sheath flow combine. The focused sample suspension exits a capillary tube and enters a larger capillary tube that has an annular flow of sheath fluid. The two fluids have identical viscosities and after a short laminar shear layer, the two liquids form a combined Poiseuille flow in the larger capillary. It is critical to preserve laminar flow in the region where the two fluids combine. Otherwise some of the microspheres in the sample flow may mix with the sheath liquid and give a degraded signal when passing through the detector region.

Figure 1 shows a schematic of the major components of the Attune acoustic focusing flow cytometer. The vertical cylinder with an open top represents the flow channel of the cytometer (not to scale). The sample suspension is introduced into the flow channel by a capillary tube; the suspension is then carried along the flow channel by a flowing sheath fluid (called focusing fluid in the Attune manual). The microspheres in the suspension pass through a region in the capillary with strong ultrasound intensity where the microspheres are effectively focused into a single file [4] which then passes through two focused laser beams, first a 405 nm beam followed by a 488 nm beam. The lasers excite microsphere fluorescence which is collected, partitioned into different wavelength ranges, and then detected with photomultiplier tubes (PMT). The digitization of the signals from the detectors is initiated by a scattering pulse from the passing microsphere. Consequently every fluorescence signal is associated with a scattering pulse whose height exceeds a preset threshold. The 405 nm and 488 nm laser paths are separated in space so that a delay occurs between the laser induced fluorescence signals originating from the two laser paths. The suspension to be analyzed is placed in a sample tube which is placed in the sample injection port (SIP) as shown in Fig. 1. The sample syringe first draws out a portion of the sample suspension and then a flow switch redirects the sample suspension from the syringe towards the capillary tube in the flow channel. A length of laminar shear, shown in Fig. 2, equilibrates the sample and sheath flows into a final configuration consisting of a core sample flow surrounded by an annular sheath flow. It is assumed that the flow is always laminar throughout the entire fluidics system (Reynolds number is of the order of 1). Laminar flow insures that there is no mixing between the sample suspension and the sheath fluid which guides the sample suspension through the laser path.

In practice, the measurements are preceded by a “performance check” of the Attune flow cytometer. The performance check is carried out by placing a suspension of special microspheres (Performance beads) in the sample tube. The performance beads are run in the cytometer as a normal sample, and the Attune software adjusts the PMT voltages and the delay between the signals associated with the two lasers, so that these values are within the expected design limits. If the performance check is passed successfully, the measurement of the analyte suspension can be initiated. The user has no control over the procedure followed in the “performance check”.

## 3. Measurement Protocol

Samples were prepared by pipetting either 1 mL or 2 mL of PBS (or another buffer) into a tube (BD Falcon 5 mL polystyrene round bottom tube 12 mm × 75 mm) containing the Trucount polystyrene microspheres with a diameter of 4.2 µm. The tube was gently mixed (Daigger Vortex Genie 2) and placed into the SIP. In most cases, the Attune was set to deliver a volume of 200 µL from the sample syringe at a flow rate of 100 µL/min. The measurement was started by drawing 314 µL of sample suspension from the tube in the SIP. The value 314 µL was determined by the Attune software and was not under control of the user (it was assumed that the draw sequence eliminated all biases due to “dead” volume of the various fluidics components). The measurement was always started in the “run” mode, and switched over to the “record mode” after about 10 µL of the sample suspension have passed. This procedure was used to minimize transient effects. The threshold of the forward scattering (FS) detector was placed in the “or” mode while the side scattering and fluorescence detectors were placed in the “ignore” mode. This required that the FS signal in a recorded event was above threshold. The amplitudes of the side scattering and fluorescence signals were localized near well-defined peak values so that ignoring a threshold, which is well below the peak value, did not bias the acquisition of “true” (near the peak) signals. The threshold settings were not expected to introduce significant uncertainty since with all thresholds set to “and” mode the number of events decreased by only 2 %, and could be rationalized as a decrease in detected electronic noise pulses. The measurements described below consisted of a sequence of approximately 10,000 scattering and fluorescence events associated with individual microspheres which passed sequentially through the laser interrogation plane.

In the following, we describe procedures to determine the number of events, and to estimate the volume associated with these events. The concentration is defined as the ratio of events and the volume. Several buffers were tried as well as a more detailed examination of the distribution of events in a gate. The objective was to find conditions which minimize systematic uncertainties.

## 4. Measurement of the Number of Events

**Fig. 3:**
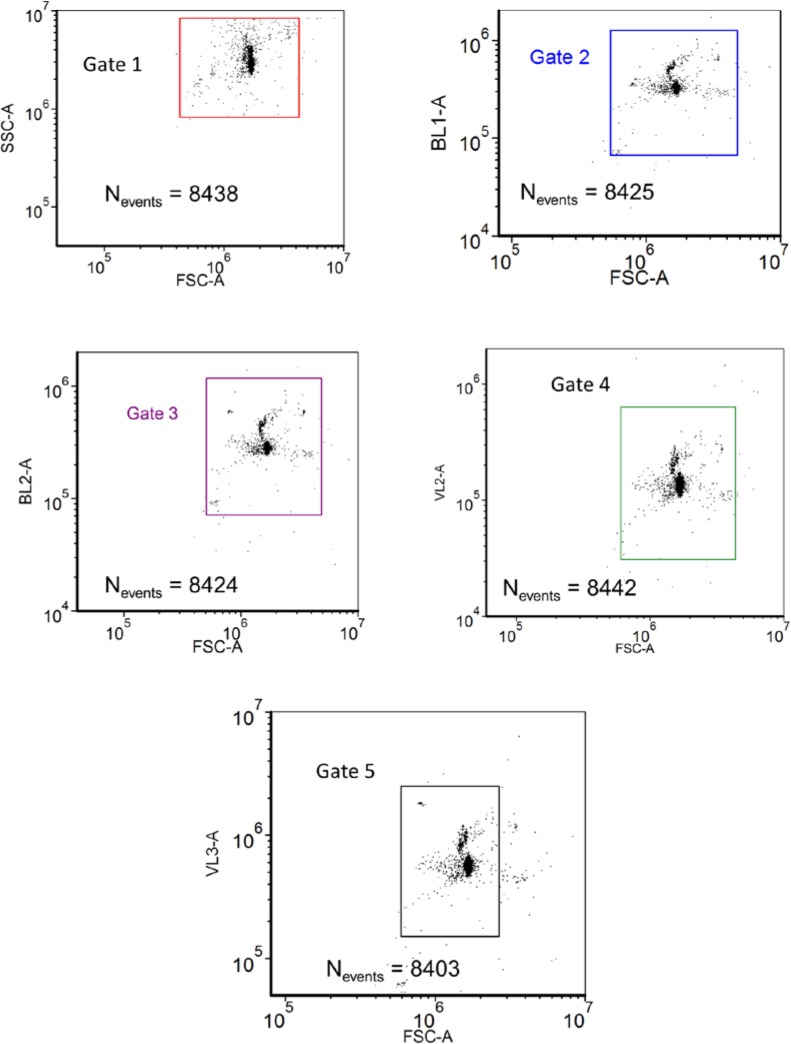
Example of the analysis which yields an estimate of the number of microspheres passing through the laser interrogation region. Each dot (event) in any of the five dot plots stands for the simultaneous occurrence of two signals denoted by the x and y axis of the dot plot. The gate in each dot plot is used to define the region used to count the total number of dots for that signal pair. The symbol N_events_ in Fig. 3 gives the number of dots (events) in each gate. The number of events is relatively constant over the signal pairs. The average of the five numbers in the second column is set equal to the number of microspheres passing through the laser interrogation region.

An event occurred whenever the signal in the forward scattering detector (associated with the 405 nm laser) exceeded a threshold value. When this happened, signals from all detectors were digitized for a preset period of time. The digitized signals were analyzed, and the area, amplitude, and width of the digitized signals (assumed to be pulses) were recorded in real time. The sequence of such recordings, taken for a preset sample volume, constituted a measurement. The analysis was performed using FCS Express V3™ [6] software by plotting the pulse area of the signal associated with one detector versus the pulse area of the signal associated with a second detector. The pulse area was chosen because it used most of the information contained in the signal pulse. Six independent signals were chosen for the determination of the number of events. The six signals were FS, side scattering (SS), the first two fluorescence channels associated with the blue laser called BL1 and BL2, and the last two fluorescence channels associated with the violet laser called VL2 and VL3. Each of the six signals originated from an independent detector responding to a different set of photons, hence the response of each detector was physically independent of the responses of the other detectors (although all signals depended on the FS signal for triggering). The FS and SS signals originated from the scattering process associated with the laser wavelength; therefore in determining the events we grouped the two scattering channels into a FS-SS density plot. This provided a possibility to discriminate between single and double microsphere passage. Similarly the four independent fluorescence signals were grouped with the FS signal to provide four density plots (dot plots) BL1-FS, BL2-FS, VL2-FS, and VL3-FS. Grouping the fluorescence signals with the FS signal recognized the primacy of the FS trigger and provided another opportunity to detect multi-sphere passages. An example of the five dot plots is shown in Fig. 3. The symbols FSC-A and SSC-A stand for forward scattering channel pulse area and side scattering channel pulse area respectively. The symbols BL1-A and BL2-A stand for blue laser fluorescence channel 1 and blue laser fluorescence channel 2 pulse areas respectively. Similar designation applies to the signals associated with the violet laser. A single dot in each of the five plots in Fig. 3 shows the location of the pulse areas of the corresponding two signals associated with that event. Approximately 8,400 events were collected for each measurement and are displayed in the five plots shown in Fig. 3. The rectangle or polygon in each plot shows the “gate” in that plot used to define the events. The event number is given by the value of N_events_. Note that the number of events in gates 1, 2, 3, 4, and 5 are almost identical as shown by the values of N_events_ associated with each plot. This means that whenever FS triggered, signals were also present in all of the other detectors. The number of events in each gate can be made larger by increasing the size of the gate. However, there is minimal change in the number of events once the gate size is as shown in the Fig. 3. Note that there are almost no low level signals (not shown in Fig. 3) suggesting that the threshold requirements eliminated most of the electronic noise. The values of N_events_ in Fig. 3 was generated using the FCS Express V3™ software. The mean value of the events in gates 1 thru 5 was 8426 and the standard deviation (SD) of the five values was 15. The SD gives an uncertainty estimate associated with gate size and placement. The five numbers do not reflect the random fluctuation in the number of microspheres passing through the laser beams (Poisson statistics) during a measurement. Although the five signals are physically distinct, they are correlated via the common interaction of the microsphere with the laser beam. The best estimate of the concentration uncertainty due to Poisson counting statistics can be obtained by simply taking the square root of the average number of events and dividing by the volume. For the data in Fig. 3, the expected relative standard deviation of the concentration due to fluctuation in the number of events is about 1.1 %, which is significantly larger than the uncertainty due to gate geometry. In the following we discuss other sources of uncertainty such as settling and aggregation, as well as the uncertainty in the volume. Table 1 gives the results obtained from a sequence of repetitions of the measurement under identical conditions. The first column entitled “sample 1” gives the results obtained from data shown in Fig. 3. The mean of the means of each sample is 8,750 with a SD of 220 or 2.5 % relative uncertainty. Therefore the preparation of the sample contributes a significant uncertainty in addition to the uncertainties due to gate placement and counting statistics. In the following we describe the calibration of the syringe volume and discuss further uncertainties.

## 5. Calibration of Syringe Volume

**Fig. 4:**
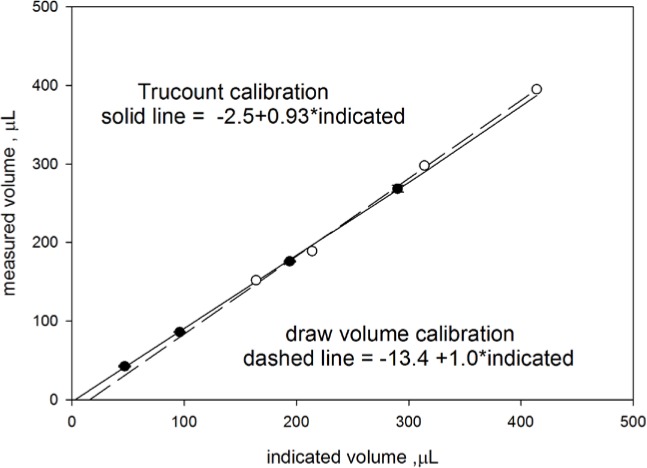
Volume calibration of the syringe used to deliver a measured amount of suspension. The open circles were obtained by weighing the sample tube in the SIP before and after a draw by the syringe. The Attune software gave the volume of the drawn sample (indicated volume) which was compared to the difference in weight of the tube in the SIP (measured volume). The dashed line is the best linear fit to the open circles. The solid circles give the volume obtained by counting the number of microspheres in a sample of known concentration (Trucount microsphere count standard was used as the reference). The solid line gives the best linear fit. In the region between 200 µL and 300 µL, the two calibration lines are practically indistinguishable.

The sample syringe delivered a measured volume of the suspension determined by the inner diameter of the syringe and the travel of the piston. The value of the delivered volume was used to calculate the concentration. The same syringe was used to draw a volume of the sample suspension contained in the tube placed in the SIP. In order to account for the volume of the tubing in the sample path, the instrument was configured to draw approximately 114 µL more than it delivers. Since the same syringe was used to draw the sample and deliver the sample, it was possible to calibrate the volume of the syringe using the capability to draw a specified volume of the sample. The calibration was performed by measuring the mass of the sample tube before the draw and after the draw. The difference of the two masses was divided by the density of water (0.99777 g/mL at 22.5 °C) to yield the drawn volume. The open circles in Fig. 4 show the results for a series of four sequential draws, and the dashed line gives the best linear representation of the data. The slope of the linear relationship is effectively 1 as expected from conservation of mass; the offset is −13.4 µL.

An alternate volume calibration was performed using a tube of Trucount microspheres (TC) which was purported to contain 50,600 microspheres. The TC microspheres were resuspended in 2 mL of PBS+0.5 % BSA buffer. A preset (indicated by software) volume of the suspension was delivered to the laser interrogation region and the number of scattering and fluorescence events was counted. The measured volume of the suspension was calculated using the expression *V* = *N_events_*/*C* where *N_events_* is the number of recorded events, and *C* is the purported concentration of the microspheres in the tube. The solid spheres in Fig. 4 show the results for four preset (indicated) volumes of the suspension, and the solid line gives the best linear representation. The offset is −2.5 µL, and the slope of the linear relation is 0.93 ± 0.02 which is problematic since it violates strict mass conservation. The reduced slope can be rationalized as a manifestation of the properties of suspension flow in a capillary (see Sec. 6.3). The similarity of the two calibration lines shown in Fig. 4 suggests that the draw and deliver actions of the syringe are very similar. In the calculation of concentrations, as described below, the delivered volume was set equal to the indicated volume minus 13 µL.

## 6. Measurement of Suspension Concentration

The motion of the sample syringe provides a volumetric flow rate of the sample suspension, and the motion of the sheath syringe provides a volumetric flow rate of the sheath fluid. Assuming laminar flow, there is no mixing between the sample suspension and the sheath fluid. Mass conservation gives a relation between the volumetric flow rate at the laser interrogation region and the volumetric flow rates of the sample and sheath fluids in the respective syringes. It was assumed that the counting of events was coordinated by the software with the start and stop positions of the syringe piston.

**Fig. 5:**
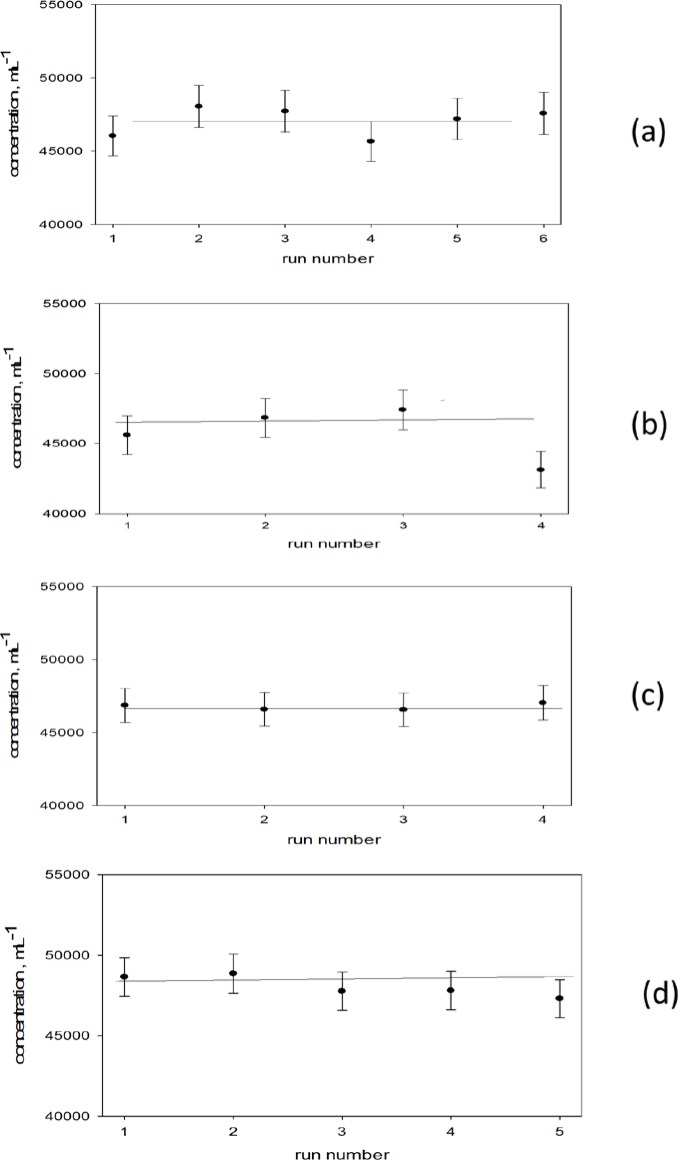
Concentration of Trucount (TC) microspheres measured in PBS under different conditions. (a) 1mL PBS into TC tube, 200 µL sample volume, 100 µL/min flow rate. (b) 2mL PBS into TC tube, 300 µL sample volume, 25 µL/min flow rate. (c) 2mL PBS into TC tube, 300 µL sample volume, 100 µL/min flow rate. (d) 1mL PBS into TC tube, 300 µL sample volume, 100 µL/min flow rate. The standard uncertainties in the data shown were about 2.5 % of the indicated values.

Examples of concentration measurements are shown in Fig. 5a, 5b, 5c, and 5d. The data points in Fig. 5a were obtained from the average values of gated events shown in Table 1. For example, the first point in Fig. 5a was equal to 46,000 mL^−1^ = 1000 µL/mL * 8426/(196 µL − 13 µL), where the indicated volume of 196 µL was obtained from the TOTALVOLUME keyword in the FCS file. Note that the indicated volume given by the keyword is slightly smaller than the sample volume (200 µL) set in the experiment control window. The difference is due to the small amount of volume which was delivered by the syringe between the time the “run” and “record” buttons were pushed. The “run” button started the motion of the syringe, and the “record” button started the recording of events in the FCS file. The samples used in Fig. 5a were obtained by pipetting 1mL PBS into the Trucount (TC) tube, and setting the Attune flow cytometer to deliver 200 µL sample volume at 100 µL/min flow rate. Under these conditions, three consecutive measurements could be taken with each TC tube, each lasting about 1.5 min.

The six points in Fig. 5a show the results of three consecutive concentration measurements on two different TC tubes (data from Table 1). The uncertainties in Fig. 5a were set equal to 3 % of the concentration obtained for each measurement. The 3 % was obtained from the standard deviation of the six concentration values shown in Fig. 5a. The four points in Fig. 5b show consecutive measurements on a third tube of TC microspheres diluted with 2 mL of PBS. (The 2 mL was taken into account when calculating the final microsphere concentration in units of mL^−1^). The sample volume was set to 300 µL, and the flow rate was set to 25 µL/min. A single measurement took about 10 min which was sufficiently long to lead to biases due to settling of microspheres. Settling appears to have a discernible but minor effect since the expected settling velocity of the TC microspheres is only 5·10^−7^ m/s [7]. The sample in Fig. 5c was prepared with 2 mL of PBS into TC tube, 300 µL sample volume and 100 µL/min flow rate. The data points in Fig. 5c should be less susceptible to biases due to settling, and indeed they show less fluctuation than those in Fig. 5b. Figure 5d shows the results of two TC tubes prepared by placing 1 mL PBS into each TC tube, a sample volume set to 300 µL, and a flow rate of 100 µL/min. (Three points for the first tube and two points for the second tube). The data in Fig. 5d are similar to those in Fig. 5a although there is slightly less fluctuations around the mean, which may be due to larger sample volume (more events counted during each measurement). The standard uncertainties in the data shown in Fig. 5 were about 2.5 % of the indicated values. The uncertainties were estimated from the actual variation in the measured concentration and included the random fluctuations in the total events (Poisson statistics), differences in gate settings, settling biases, and pipette error during dilution of the TC tubes. In the following we discuss additional sources of uncertainty due to adsorption of microspheres on the container walls, and possible aggregation of microspheres in suspension.

### 6.1 Biases Due to Adsorption of Microspheres

**Fig. 6:**
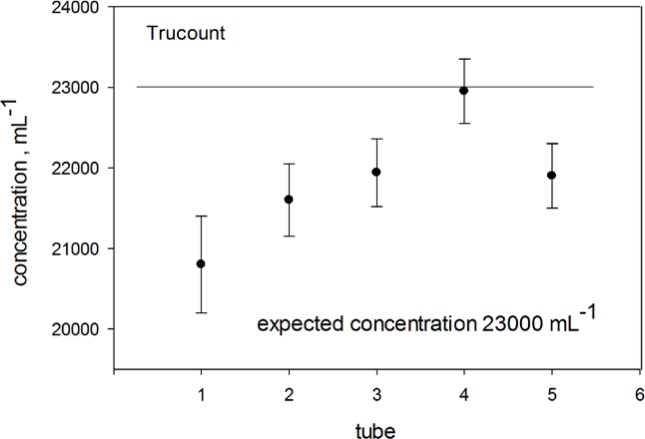
The concentration of Trucount microspheres obtained in different buffers. Tube 1 contains a PBS buffer. Tubes 2 and 3 contain PBS+0.0 5 % TWEEN 20, while tubes 4 and 5 contain a PBS+0.05 % BSA buffer. All tubes contain 2 mL of buffer and 0.2 mL of a suspension of fluorescein labeled microspheres. The standard deviation from the three consecutive concentration measurements for each sample tube was about 3 % or less. The expected concentration of Trucount microspheres is given by 50,600/2.2 as discussed in the text.

Measurements of biological samples in microfluidic devices are biased by adsorption of components of the sample suspension on surfaces of the microfluidic device. It has been demonstrated that including surfactants in the suspension minimizes the adsorption [8]. Five measurements were made on samples prepared by pipetting 2 mL of buffer and 0.2 mL of fluorescein labeled microspheres into the TC tube. Some of the samples contained surfactants. During the measurement, the sample volume was set to 300 μL, and the flow rate was set to 100 μL/min. In all of the measurements, the microsphere concentration was in the dilute region so that reduction of concentration due to adsorption of microspheres on the container surfaces should be proportional to the microsphere concentration in suspension. Since laminar flow was assumed, there was no loss of microspheres due to mixing with the sheath fluid. Adsorption equilibrium in the vial should be rapid - less than a minute. Measurements were performed in three different buffers: pure PBS (Ambion 10X PBS Buffer pH 7.4, AM9625, diluted tenfold with deionized water), PBS with 0.05 % BSA by mass, and finally PBS with 0.05 % TWEEN 20 by volume. Figure 6 shows the effect of buffer on the measured TC concentration. The horizontal axis is labeled “tube” with each tube containing a buffer. Tube 1 contained PBS. Tubes 2 and 3 had PBS plus 0.05 % TWEEN 20, with slight mixing (with vortex mixer) of tube 3 prior to measurement. Tubes 4 and 5 contained PBS plus BSA, with slight mixing of tube 5 prior to measurement. The concentration measured in tube 1 was consistent with the average value of 21,000 mL^−1^ obtained from the measurements shown in Fig. 5. The value of the concentration of TC microspheres in tube 4 (PBS+BSA buffer) was significantly higher than the value in PBS alone. The concentration measured in PBS plus 0.05 % TWEEN 20 (tubes 2 and 3) were statistically indistinguishable from the values measured in PBS+BSA. Effects of mixing were also statistically indistinguishable. Since mixing enhances the dissolution of the TC pellet as well as the homogeneity of the microsphere suspension, mixing should be included as part of the measurement protocol. To emphasize the importance of adding BSA to the TC suspension, concentration measurements were performed on three tubes of TC suspended in 2 mL of PBS and three tubes of TC suspended in 2 mL of PBS+0.05 % BSA. The resulting concentration values were (23290, 23050, 23440) and (24490, 24780, 24630) respectively. All TC tubes were from the same lot (Lot #13254). A student t-test on the two sets of concentration values gave a 95 % confidence level that the means of the two sets of concentration values were significantly different. The results are consistent with previously reported effects of buffer composition on the measured microsphere concentration [9]. The measurements also suggest that the uncertainty in the number of microspheres in any given tube from the same lot is of the order of a few hundred microspheres.

### 6.2 Aggregation

**Fig. 7:**
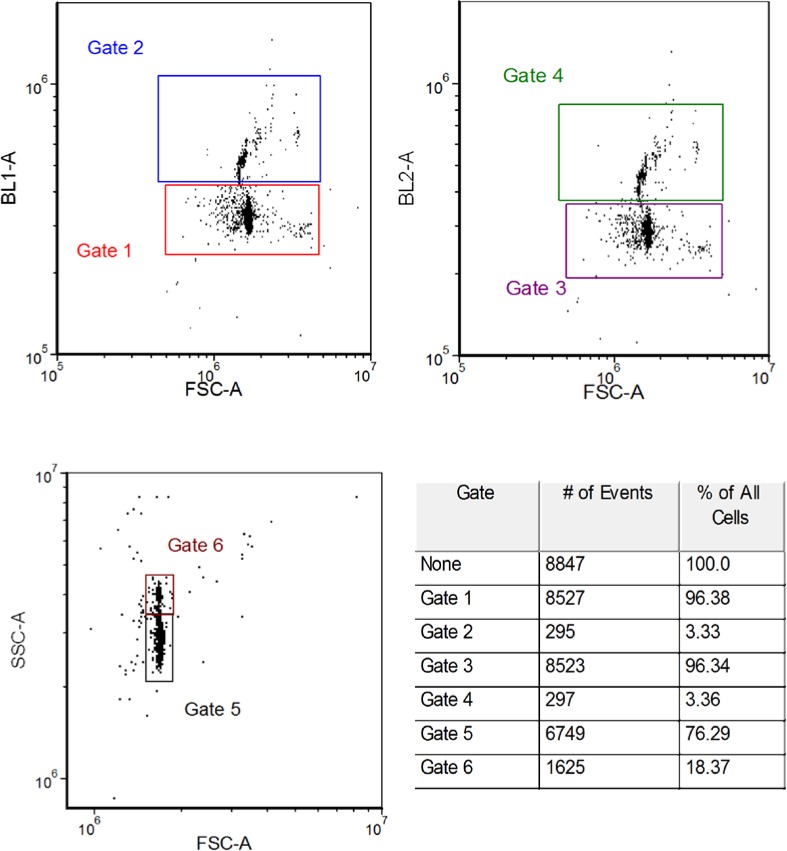
Detailed examination of the dot plots in the region of highest dot density. The major population, most likely due to single microspheres, is contained in gates 1, 3, and 5, while a minor population is located in gates 2, 4, and 6. The minor population may be due to aggregates of microspheres. The two populations are relatively distinct in the fluorescence channels, and poorly defined in the scattering channels.

Figure 7 shows a magnified view of the dot plot for BL1-FS and BL2-FS signal pairs. The sample was a suspension of TC in PBS. The examination of the dot plots show that the distribution of the fluorescence areas in the BL1-FS dot plot has a major peak (96.4 %) and a minor peak (3.3 %) at higher fluorescence intensity values. Assuming that the minor peak at higher fluorescence is due to microsphere doublets, the number of events in the small peak should be doubled and combined with the number of events in the large peak. The table in Fig. 7 shows the number of events in the major and minor peaks. When the events in the two peaks were counted as single microspheres, the average concentration was 47,400 mL^−1^. When the events in the major peak were combined with twice the number of events in the minor peak the microsphere concentration was 49,000 mL^−1^. Therefore the splitting of the total dot plot into singlets and doublets leads to an increase of concentration by about 2 %. However, the identification of the minor peak as doublets is not consistent with scattering signals shown in the FS-SS dot plot in Fig. 7. The FS axis doesn’t show a second population as would be expected if there was a small portion of larger microsphere aggregates in the suspension. The SS axis may be divided into two populations as shown in the dot plot by gates 5 and gates 6. However, the ratio of the two populations is 76 %/18 % which is not consistent with the ratio of the two fluorescence peak populations, 96 %/3 %. Imposing the requirement that the events shown in the SS vs FS plot in Fig. 7 also fall in either gate 1 or gate 2 in the BL1 vs FS plot in Fig. 7, shows that there is no correlation between the distribution of events in the SS vs FC plot and the distribution of events in the BL1 vs FC plot. It may be that all of the microspheres have the same size, and only the loading of dye is larger in a small population of the microspheres. In what follows, it was assumed that the events in the dot plots were due to single microspheres. The possible undercounting of microspheres could be a source of a systematic uncertainty.

### 6.3 Artifacts Due to Characteristics of Flow in Capillaries

The difference in the slope of the two volume calibration curves shown in Fig. 4 suggests that there may be subtle differences in the physical basis of the two calibration methods. For example, the laminar flow in a capillary is characterized by a quadratic dependence of the flow velocity on radial distance. The non-uniform flow leads to a migration of the microspheres towards the center of the capillary tube [10]. The correlated increase in both velocity and the local microsphere concentration at the center of the capillary tube results in a flow which conserves the average suspension volume flux while at the same time leads to a reduced average microsphere concentration inside the capillary tube [11]. The expected reduction is given by Eq. (5) in Ref. [11] which could arise due to transient effects during the inception of the flow. Another explanation could be a loss of microspheres in the channel flow due to hold up in the transitions between channel components or adsorption to channel surfaces. The difference between the slopes of the two volume calibrations has to be resolved before the measurement of the microsphere concentration with the Attune flow cytometer can be characterized as absolute.

### 6.4 Suggested Measurement Procedure

**Fig. 8:**
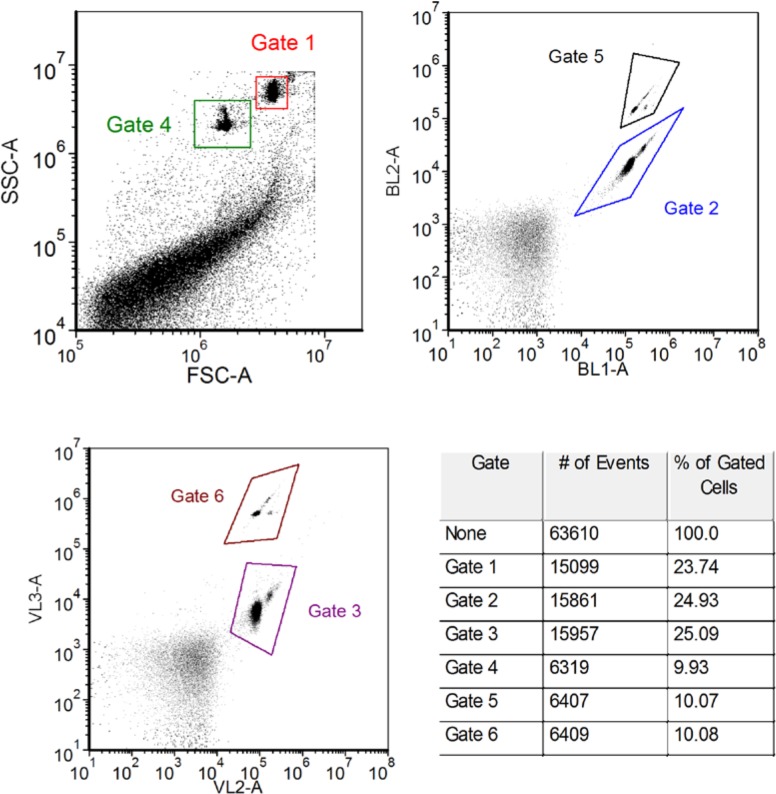
The data for a sample containing Trucount and fluorescein labeled microspheres (RM 8640) in PBS+0.05 % BSA. Gates 4, 5, and 6 identify events associated with the Trucount, and gates 1, 2, and 3 identify RM 8640 events. The second column of the table shows the event number for each of the gates shown in the dot plots.

The suggested procedure for the measurement of the concentration of microspheres is to use a buffer PBS+0.05 % BSA, accumulate data for a set volume of 200 µL to 300 µL, and reduce the indicated volume by 13 µL when performing the concentration calculation. (The number 13 µL is the result of a volume calibration performed on a specific instrument and may not reflect a general property of all Attune flow cytometers). Figure 8 gives an example of the procedure applied to the measurement of the concentration of a mixture of Trucount (TC) and fluorescein labeled microspheres (expired RM 8640). The sample was prepared by pipetting 2 mL of PBS+0.05 % BSA buffer, and 0.2 mL of RM 8640 suspension into a tube of TC microspheres. The two populations in the dot plots correspond to the TC (Gates 4, 5, 6) and RM 8640 (gates 1, 2, 3). The populations were identified by measuring each microsphere population separately. The indicated volume of 289 µL was given by the Attune software. The event numbers shown in the table in Fig. 8 were calculated by setting the gates as shown in Fig. 8. The resulting TC concentration was 23,100 mL^−1^ which compares favorably with the expected value of 23,000 mL^−1^. The RM concentration was 56,600 mL^−1^ using the syringe volume calibration method described above. The RM concentration was also evaluated relative to the purported concentration of TC and given by the relation *C_RM_* = (*N_RM_* /*N_TC_*)23000 where *N_RM_* and *N_TC_* are given by the averaged event numbers in the table in Fig. 8. The concentration using the relative method was 56,500 mL^−1^. All uncertainties were 2.5 %.

## 7. Conclusion

The Trucount tubes contain about 50,600 microspheres while the measurements with the Attune flow cytometer (with PBS+0.05 % BSA buffer and volume calibration) gave about 50,800 ± 1500 mL^−1^. Therefore the measurements with the Attune flow cytometer can provide reliable values of the microsphere concentration. Possible source of uncertainty were adsorption, aggregation, settling, and volume calibration. The adsorption of microspheres was reduced by including an adsorption blocking agent (BSA) in the suspension buffer. Settling was reduced by mixing the suspension, however random fluctuations in microsphere concentration near the sampling tube in the SIP are inevitable. Volume delivered by syringe was calibrated by either directly weighing the mass drawn by the syringe or using a suspension of known concentration. Finally aggregation was minimized by gentle mixing. Overall an uncertainty in the measured concentration of 4 % could be rationalized. Since a calibration is needed to validate the indicated volume given by the Attune software, it would be useful if the Attune provided a capability to calibrate the volume delivered by the sample syringe (rather than volume drawn by the syringe). In addition, a gentle stirrer in the SIP would insure better mixing and minimize settling. With proper attention given to the buffer and the volume calibration, the flow cytometer with a volumetric sample delivery is well adapted to the measurement of microsphere concentration.

## Figures and Tables

**Table 1:** Number of gated events from repetitive measurements

Gate	Sample 1	Sample 2	Sample 3	Sample 4	Sample 5	Sample 6
FS-SS	8438	9004	8896	8542	8778	8906
BL1-FS	8425	8973	8866	8534	8775	8887
BL2-FS	8424	8966	8860	8524	8769	8884
VL2-FS	8442	9018	8904	8568	8806	8935
VL3-FS	8403	8962	8854	8524	8769	8878
						
Sample mean	8426	8984	8876	8538	8779	8898

## Footnotes

1 Certain commercial equipment, instruments, and materials are identified in this paper to foster understanding. Such identification does not imply recommendation or endorsement by the National Institute of Standards and Technology.
